# Impact of two bundles on central catheter-related bloodstream infection in critically ill patients^1^

**DOI:** 10.1590/1518-8345.2190.2951

**Published:** 2017-12-04

**Authors:** Cristobal Felipe Padilla Fortunatti

**Affiliations:** 2 Student in Master’s degree, Escuela de Enfermería, Pontificia Universidad Católica de Chile, Santiago, Chile. RN, Hospital Clinico Red de Salud UC - CHRISTUS, Santiago, Chile.

**Keywords:** Cross Infection, Intensive Care Units, Prevention and Control, Adult, Central Venous Catheterization, Quality Improvement

## Abstract

**Objective:**

To evaluate the impact of the implementation of insertion and maintenance bundles on the rates of catheter-related bloodstream infection in an intensive care unit.

**Method:**

This is a quasi-experimental, before-and-after study with a non-equivalent control group. During a six-month period, insertion and maintenance bundles for the central venous catheters were implemented. Supervision guidelines were developed to assess compliance with the bundle and catheter characteristics.

**Results:**

A total of 444 central catheters corresponding to 390 patients were observed, of which 68.7% were inserted in the unit. The maintenance and insertion bundles reached 62.9% and 94.7% compliance, respectively, and 50.7% of the insertions were supervised. It was possible to observe a 54.5% decrease in the rate of central catheter infection (3.48 vs 1.52 x 1000 days/catheter, p<0.05) when compared with the control group.

**Conclusion:**

The simultaneous implementation of insertion and maintenance bundles has a positive impact on the reduction of catheter-related bloodstream infection; therefore it is an efficient alternative to improve the quality and safety of care in high complexity units.

## Introduction

Intensive care units (ICUs) are highly qualified to care and treat patients at risk through invasive therapy, procedures and devices such as the central venous catheter (CVC). The CVC is one of the most common devices in the ICU, since it is used to monitor hemodynamics and deliver vasoactive drugs, antibiotics and total parenteral nutrition ^1^ ^-^ ^2^. Despite its benefits, the CVC can lead to mechanical or infectious complications. The latter are more frequent and have greater impact on the patient ^3^ ^-^ ^4^. Catheter-related bloodstream infection (CRBSI) is a complication that can be related to increases in costs, length of stay and morbidity and mortality rates, especially among ICU patients^5^ ^-^ ^7^. A recent analysis showed a 2.75-fold increase in hospital mortality and a 2.15-fold increase in CRBSI on ICU patients ^8^. Likewise, a study from Argentina found that CRBSI was associated with an additional cost of almost $5,000 and an increase in hospital stay of 12 days for each episode^9^. 

There are several risk factors associated with CRBSI, such as: duration of catheterization, number of lumens, femoral access site, excessive manipulation of the CVC, total parenteral nutrition, bacterial colonization at the insertion site, prolonged hospitalization, and others ^10^ ^-^ ^12^. There are several strategies to prevent CRBSI, and bundles are recognized as one of the most used and most effective for the reduction of CRBSI ^10^ ^,^ ^13^. Bundles can be defined as the systematic implementation of a set of evidence-based practices, usually three to five, that, when performed properly and collectively, can improve patient outcomes^13^. Research on CRBSI prevention demonstrated the effectiveness of bundles, which reduce the incidence of CRBSI by up to 80% ^5^ ^-^ ^6^ ^,^ ^14^, reaching a rate of 0 in some cases^4^ ^,^ ^15^. 

The bundles for the prevention of CRBSI include good hand hygiene, preparation of the skin with chlorhexidine gluconate, preference for the subclavian vein, maximal sterile barriers, and daily assessment of the need for the CVC ^16^. Thus, basic infection control practices can significantly reduce the incidence of CRBSI, reducing the rate of 6.5 to 46 cases per 1000 CVC days ^17^.

In order to promote quality and safety in the care process, health institutions had to explore and adopt practices to minimize risks to patients. An unusual increase in the number of CRBSI cases was observed in the unit under study on the first semester of 2015. This motivated the creation of a quality assurance plan to reduce and prevent these events, including the implementation of a CRBSI bundle. The objective of this study was to evaluate the impact of the implementation of CVC insertion and maintenance bundles on CRBSI rates in a medical-surgical ICU (MSICU).

## Method

Quasi-experimental study before-and-after intervention, conducted with nonequivalent control group in the context of the quality assurance plan of a MSICU for adults. The MSICU has 32 beds, divided into high and medium complexity care, belonging to a teaching hospital in Santiago, Chile. This MSICU has 24-hour on-site intensivists, and fellows of the same specialty or others, who take turns throughout the year. As for the nursing team, the usual ratio is 1:2 to 1:3, for both nurses and nursing technicians. Most of the MSICU CVCs (temporary, hemodialysis or peripheral insertion) are inserted by intensivists; however, the unit also receives patients whose CVC was inserted in other hospital services or that were transferred from other health care facilities. 

Regarding the device-associated care, the CVC dressing is valid for seven days if it is possible to visualize the insertion site, which must remain clean and intact. If the insertion site is not visible, the dressing should be changed within 48 hours. In addition, 2% chlorhexidine-impregnated dressings are available to be used at the discretion of the nurse. The needleless connectors are changed every 72 hours or when the infusion pump is changed. Total parenteral nutrition is administered through an exclusive lumen catheter.

Between January and June 2016, insertion and maintenance bundles, each composed of 3 measures, were simultaneously implemented to prevent CRBSI. The insertion bundle consisted of operator and assistant hand hygiene, preparation of the skin with 2% chlorhexidine soap and use of maximal sterile barriers for the operator and the patient. The maintenance bundle included: daily evaluation of the need for the CVC, verification of the CVC insertion site and dressing and daily bathing with 2% chlorhexidine gluconate. 

For the insertion bundle, insertion of the CVC in the unit under study was considered as inclusion criterion. CVCs that, due to urgent need for vascular access, did not comply with the insertion measures were excluded. For the maintenance bundle, all the temporary CVCs with permanence of at least 24 hours in the MSICU were included. The CVCs used for renal replacement therapy were excluded from this bundle, since they are not handled by the nurses of the MSICU.

For data collection, supervision guidelines for each bundle were developed in cooperation with the local Committee for the Prevention and Control of Healthcare-Associated Infections. For the insertion bundle, the guidelines included information about the type of ICU where the CVC was inserted (medical/surgical), number of lumens, insertion site and compliance with each measure. For the maintenance bundle, in addition to the previous variables, the place where the CVC was inserted, whether in the MSICU or in another unit, was also included. The data related to the insertion bundle were collected at the time of insertion of the CVC by the nurse in charge of the patient, while for the maintenance bundle a nurse was assigned to retrospectively evaluate compliance with the measures at each period, based in the patient’s clinical records. 

Before the intervention, the MSICU health team received training on the measures included in each bundle and how to perform them. The physicians were instructed on the maximal sterile barriers, since they involved substantial changes in the usual clinical practice and assessment of the need to maintain the CVC in partnership with the nurse in charge of the patient. For the nurses, the maintenance bundle records were reinforced in the nursing notes, and for the nursing technicians the training was focused on the technique for skin preparation for the insertion of the CVC. During this period, the supervision guidelines were tested to familiarize health professionals and to make the necessary adjustments without significant changes.

In addition, a series of tests was performed in the period prior to the beginning of the intervention and it was possible to identify and correct operational aspects regarding its implementation. Compliance statistics were reported every month to the entire MSICU team to identify opportunities for improvement and provide positive feedback, if appropriate.

A nonequivalent control group composed of patients who had a CVC during the same period (January - June) of 2015 was used to evaluate the impact of the intervention, following the same inclusion and exclusion criteria of the intervention group. Likewise, data related to the CRBSI rate, the mean duration of the CVC and the number of CRBSI cases during that period were used. The CRBSI case definition was done independently by the local Committee for the Prevention and Control of Healthcare-Associated Infections. 

The compliance with the bundles was expressed in percentages. In order to analyze the variables of interest (mean duration of CVC, number of CRBSI cases and CRBSI rate), the Student ‘s T test, the Chi-square test and the Fisher’ s exact test were used as required. The statistical software GraphPad (GraphPad Software, La Jolla California USA, www.graphpad.com) was used for the statistical analysis. The p value <0.05 was considered statistically significant.

Both the data collection and the analysis were performed retrospectively, without the identification of clinical data, which is why the exemption of informed consent was requested and accepted by the Research Ethics Committee of the Medical School of the Pontifical Catholic University of Chile. 

## Results

During the intervention period, a total of 444 CVCs were observed, corresponding to 390 critical patients, which totaled 2629 CVC days. Table 1 shows the general characteristics of the CVCs. Triple-lumen CVC (72.5%) and insertion in the jugular vein (46.8%) were more frequent. Most of the CVCs assessed were inserted in the MSICU (68.7%) and 38.3% were removed while the patient remained in the MSICU. 

**Table 1:** Distribution of frequencies of CVC characteristics during the intervention period. Santiago, Chile, 2016

	**n**	**%**
Type of ICU*		
**Medical**	**215**	**48.4**
**Surgical**	**229**	**51.6**
**Number of catheter lumens**		
**5 lumens**	**35**	**7.9**
**3 lumens**	**322**	**72.5**
**2 lumens**	**51**	**11.5**
**Other**	**36**	**8.1**
**Insertion site**		
**Subclavian**	**171**	**38.5**
**Jugular**	**208**	**46.8**
**Femoral**	**14**	**3.2**
**Antecubital**	**51**	**11.5**
**Origin of the CVC** ^**†**^		
Inserted in the ICU*	**305**	**68.7**
Inserted outside the ICU*	**139**	**31.3**
**Outcome of the CVC** ^**†**^		
Transferred from ICU*	**237**	**53.4**
Removed in the ICU*	**170**	**38.3**
**Patient died with CVC** ^**†**^	**37**	**8.3**

*ICU: Intensive care unit; †CVC: Central venous catheter

Regarding the insertion bundle, there were records in 51.5% of the CVCs inserted in the MSICU (n = 157). Overall compliance was 93.8%, with greater compliance to hand hygiene and skin preparation (100%), while the use of maximal sterile barriers reached 93.8% compliance.

**Figure 1:**
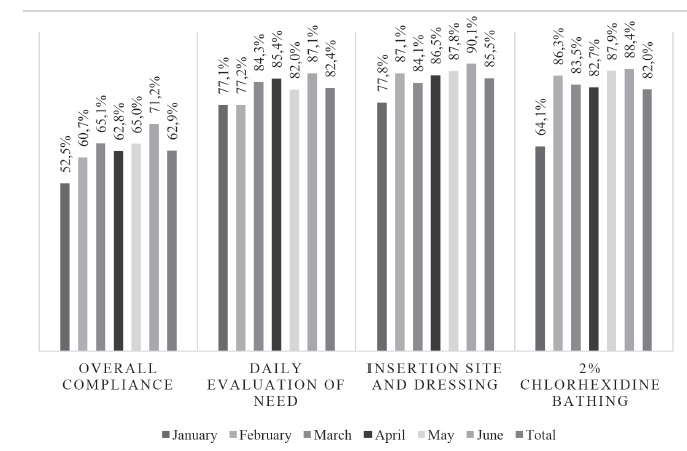
Description of monthly compliance with the CVC* maintenance bundle. Santiago, Chile, 2016

In Figure 1 it is possible to observe that the maintenance bundle reached an overall compliance of 62.9%, obtaining its minimum value at the beginning of the intervention (52.5%) and the maximum at the end (71.2%). Regarding the final compliance of each measure of the bundle, the evaluation of the need for the CVC reached approximately 82.4%, inspection of the insertion site and dressing reached 85.5%, and 2% chlorhexidine bathing reached 82.0%.

*Central venous catheter

**Figure 2:**
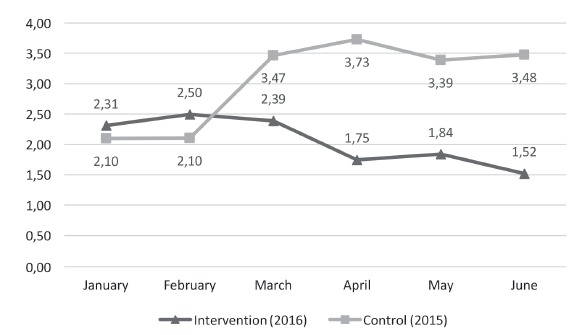
Comparison of the accumulated rate of CRBSI* on the intervention and control periods (x 1000 CVC days^†^). Santiago, Chile, 2016

Figure 2 shows the evolution of the CRBSI rate during the control and intervention periods. At the beginning of the control period, CRBSI rates were lower than during the intervention period (2.10 vs. 2.36 x 1000 CVC days); however, it is possible to observe that in the latter period the rates began to decrease, becoming smaller (1.52 x 1000 CVC days) than in the control period (3.48 x 1000 CVC days).

*Catheter-related bloodstream infection; ^†^Central Venous Catheter

Regarding the impact of the bundles, Table 2 shows a significant decrease of 28.9% in the mean duration of the CVC, a 60.0% decrease in the number of CRBSI cases and a 54.5% decrease in the CRBSI rate compared to the control period.

**Table 2:** Comparison of the variables of interest during the periods of control and intervention. Santiago, Chile, 2016

	**Control Period** **(1’ 2015)**	**Intervention Period** **(1’ 2016)**	**Variation** **(%)**
CVC days* **(n)**	**2877**	**2629**	**- 8.6**
Mean duration of CVC* **(days)**	**8.3**	**5.9**	**- 28.9** ^**†**^
**No. of CRBSI** ^**‡**^ **(n)**	**10**	**4**	**- 60.0** ^§^
Accumulated CRBSI* rate (x 1000 CVC days*)	**3.48**	**1.52**	**- 54.5** ^**§**^

*Central venous catheter; ^†^p < 0.01; ^‡^Catheter-related bloodstream infection; ^§^p < 0,05

During the intervention period, there were four cases of CRBSI, all corresponding to CVCs inserted in the ICU. In addition, no case of CRBSI was reported in patients transferred with their CVC in situ, which could have been later attributed to the ICU.

## Discussion

The objective of this study was to evaluate the impact of the simultaneous implementation of insertion and maintenance bundles on CRBSI rates in the ICU of a university hospital.

The number of records of the insertion bundle and the percentage of efficacy of the maintenance bundle deserve consideration, since they suggest the existence of other factors that can have influenced the reduction of CRBSI. There are several studies that demonstrate the effectiveness of bundles for the prevention of CRBSI; however, there is a high heterogeneity in the measures included in each bundle. In the literature, most of the times there is only one bundle, with measures focused mainly on insertion, including a daily evaluation of the need for CVC as a single maintenance measure ^4^ ^,^ ^16^.

In this study, the simultaneous implementation of two bundles may have compensated for the lack of reports on the insertion bundle and the low compliance with the maintenance bundle. The level of compliance with the maintenance bundle was low compared to other studies ^18^ ^-^ ^19^. A 95% adherence is suggested in order to achieve a substantial reduction in CRBSI rates, which is usually not achieved ^20^. The maintenance bundle, on the other hand, reached high compliance levels for each individual measure and included the daily evaluation of the CVC insertion site, a measure that is not frequent, despite its presence in the bundles that led to a decrease in the CRBSI rate ^18^ ^-^ ^19^.

Another factor that may have influenced the reduction of CRBSI is the inclusion of daily bathing with 2% chlorhexidine gluconate, which is normally not included in the bundle measures ^6^ ^,^ ^10^. The use of chlorhexidine instead of normal soap is based on reducing the bacterial colonization on the patient’s skin, which can enter the bloodstream via the extraluminal CVC route ^21^. The chlorhexidine cleaning is recommended when the basic measures for the prevention of CRBSI did not achieve the results expected; however, its use is justified when the CRBSI rates are above institutional limits ^11^. 

The low level of compliance with the maintenance bundle, especially during the first half of the intervention period, can be attributed to the inclusion and progressive knowledge of new measures in an ICU where the bundle methodology was first implemented. The incorporation of new practices can be complex for health teams, who face little familiarity with the clinical guidelines, lack of resources and low level of self-efficacy as obstacles to the implementation and execution of these practices ^1^. However, almost 80% of the ICU team was trained on the bundles and received monthly suggestions and comments regarding their effectiveness.

In addition, the first three months of the control period and of the intervention period coincided with the arrival of substitute staff in the unit and especially in nursing, which was not familiar with the local practices of CRBSI prevention protocol. Some studies have associated the presence of temporary nurses with an increased risk of CRBSI in ICUs ^22^, reaching an increase of up to 3.8 fold under certain conditions when compared to the work of regular employees ^23^. Similarly, despite the high level of compliance, there were a low number of reports of the insertion bundle, which could be attributed to the workload of the nurses responsible for supervising and recording this procedure. 

Regarding the 2% chlorhexidine bathing, a differential analysis was not performed on cases where the bathing did not occur due to the patient’s condition (hemodynamic instability, recent extubation or intubation, emergency procedures, etc.) or if the person question rejected the cleaning. The low compliance in the first month can be attributed to an initial lack of knowledge about this measure and the correct method of recording it.

Among the strengths of this intervention is the simultaneous application of the bundles in two procedures that are critical for the prevention of CRBSI, insertion and maintenance. In addition, the fact that a nurse supervises compliance with the insertion bundle ensures the accuracy of the report.

Even with its positive impact, this study has several limitations. First, the quasi-experimental design makes it impossible to relate efficacy and causality only to the intervention. Likewise, conducting the study in a single hospital center in Chile limits the external validity of the results in ICUs with similar characteristics. Also, because it was an initiative within the context of a local quality assurance plan, data on the risk factors for CRBSI in the patients involved were not collected, which makes it impossible to determine if both groups were comparable.

Unlike the insertion bundle, the effectiveness of the maintenance bundle was based on the assessment of clinical records, with no verification of the accuracy of such data. In addition, the monitoring and supervision periods of the bundles did not allow evaluating the compliance and the impact on the CRBSI on medium-term. Although the literature reports similar periods of surveillance ^16^ ^,^ ^18^ ^,^ ^24^, other studies have described periods longer than 6 months ^7^ ^,^ ^25^ ^-^ ^26^. 

## Conclusion

The implementation of a strategy based on the simultaneous application of insertion and maintenance bundles has a positive impact on the reduction of CRBSI in critically ill patients. The low percentage of insertion bundle records and the moderate compliance to the maintenance bundle suggest the presence of other factors or a synergistic effect of both bundles on the decrease of CRBSI. The intensive care nurses play a fundamental role in the critical processes that determine the occurrence of CRBSI, therefore they are workers that assure the quality and safety of care for the critically ill patient.
